# Developmental Issues in Underage Drinking Research

**Published:** 2004

**Keywords:** underage drinking, adolescence, growth and development, biological maturation, psychological development, brain, cognitive development, risk factors, protective factors, social adjustment, peer group, gender differences, intervention, prevention, statistical modeling

## Abstract

To better understand underage drinking and how it can be prevented, research is being conducted in a wide variety of disciplines—focusing on aspects such as risk and protective factors, biological processes underlying human development, and the impact of socioenvironmental and pharmacologic influences on these mechanisms. This article examines underage drinking from a developmental perspective, which seeks to identify critical developmental periods during which interventions may be especially useful. These critical periods can provide key opportunities to redirect the course of development and alter the life course trajectory of the individual.

## Overview

A mix of many different kinds of factors underlies the development of problem drinking in adolescents. For this reason, research focusing on any one area is likely to miss the complex interactions that shape how an adolescent will respond to the availability of alcohol.

Research that takes a developmental perspective seeks to provide an understanding of behavior in the context of the changes that take place during human maturation. The developmental perspective assumes interactions that not only are complex but that change over time.

The speed and timing of development are not uniform. The biologically based ability of a person to regulate mood as well as outwardly directed behavior, for example, changes during adolescence as the brain matures. The progress of these changes can affect how well an adolescent handles the tasks of adolescence—achieving autonomy and taking on more adult roles—or whether problems arise from a mismatch of development and social pressures.

Girls and boys differ not only in the pace of physical maturation but also in how they respond to the resulting social experiences for which physical changes serve as stimuli. In ways that are different for boys and girls, the attachments children form with peers or older teens can influence their risk of involvement in potentially harmful behavior.

One of the challenges of this research is to develop theories that then can be tested using statistical models to determine how well they predict how the complex array of social, cultural, environmental, and biological factors interact to increase or reduce the risk of underage drinking.

## The Developmental Perspective

In the effort to understand underage drinking, research in many disciplines has contributed valuable information on risk and protective factors, biological processes underlying human development, and the impact of socioenviron-only mental and pharmacologic influencesin the on these mechanisms. To date, much of this diverse work has been aligned with specific disciplinary paradigms. Problematic involvement with alcohol is multicausal, however; studies conducted within any single research discipline lack the breadth required for a comprehensive approach to the elucidation of risk and protective factors and to develop improved interventions. Genetic and socioenvironmental factors act together through biological mechanisms to generate the complexities of behavior, including early-age problematic involvement with alcohol.

The developmental perspective is a life course approach to understanding behavioral problems such as underage drinking and its consequences. This perspective has evolved from relatively recent advances in the fields of developmental psychopathology, human brain development, and behavior genetics. It is set in the context of the chronology of human maturation and the multiple social and cultural systems that interact with the developing human. Like systems biology, it posits complex multidirectional and reciprocal interactions that change over time. Viewed in this way, development encompasses not only the roots of risk and resilience in maturational pathways and developmental stages but also the modulation of behavior by present circumstances (Sroufe and Rutter 1984). The developmental perspective can inform the development of strategies and opportunities to prevent adverse, health-compromising drinking outcomes. It also can shape the content and process of therapeutic interventions.

## Characteristics of Developmental Research

Developmental research is, by nature, longitudinal. There are multiple possible starting points and developmental pathways to a problematic or positive adolescent outcome. Young people who are vulnerable as preadolescents can acquire positive, health-promoting low-risk behaviors upon reaching adolescence. Others who are low risk as preadolescents can have substantial problematic involvement with alcohol in later adolescence. Cross-sectional strategies may not reveal the complex interactions of multiple causal factors with biological, cognitive, affective, psychological, and social maturational milestones in determining relevant positive and negative outcomes. This is particularly true given the variation within the population in the timing and achievement of these milestones.

A key assumption underlying developmental research is that, although maturation is progressive, it is not uniform in speed or timing. There are periods of rapid transition, reorganization, and spurts of growth (i.e., saltation), alternating with periods of quiescence and consolidation (i.e., stasis). Rapid transitions may be critical developmental periods during which the social or cultural environment most strongly influences the biology and behavior of the developing human, leading to either an adverse or positive outcome (reviewed in Greenough et al. 1987; Cicchetti and Tucker 1994). Critical developmental periods may provide key opportunities to redirect the course of development and alter the life course trajectory of the person (Masten 2004). Timing health-promoting interventions in terms of critical developmental transitions could enhance efficacy.

## Social Context

Social context seems to be particularly important in understanding and modifying human developmental trajectories. During adolescence, parental influences continue to be important, but there is a progressive increase in the influence of peers. Perhaps reflective of this altered balance, Brook and colleagues (1990) noted that family-directed alcohol and other drug abuse prevention efforts are generally more effective for children, whereas interventions involving peers are more effective for adolescents.

## Self-Regulation

Self-regulation refers to the organism’s ability to monitor and modulate internal states. In humans, it includes both the ability to modulate affect and level of arousal and the neurocognitive executive capacities to engage in goal-directed behavior. These executive cognitive capacities include the regulation of attention, planning, organization, concept formation, abstract reasoning, cognitive flexibility, self-monitoring, motor programming, and motor control (Stuss and Benson 1984). Self-regulatory behavioral capacities are refined during early adolescence as neurobiological maturation progresses and frontal brain regions mature. In parallel, early adolescence is characterized by the emergence of a social drive to establish an adult role, behave autonomously, and engage in adult decisionmaking. This social drive for autonomy and adult status is further stimulated by the mass media’s shaping of perceived social norms for adolescents.

For some adolescents, however, there may be a mismatch between the drive to assume an adult social role and the adolescents’ biologically mediated capacity to regulate internal mood states and outwardly directed behavior. Research indicates that this mismatch between social aspirations and self-regulatory abilities increases the adolescent’s vulnerability to a variety of adverse behavioral outcomes, including problematic involvement with alcohol and other drugs (Dahl 2004). To mitigate problems arising from this mismatch in cognitive-emotional abilities and social pressures, the balanced support, monitoring, and modeling of social roles by influential adults (known as social scaffolding) can help guide and protect the adolescent through this vulnerable period.

## Pubertal Timing, Social Factors, and Gender Heterogeneity

Boys and girls differ significantly in the onset, tempo, and phenomenology of physical sexual maturation. Puberty’s physical changes serve as stimuli for dynamic changes in the social experience of maturing children. These social experiences require significant psychosocial adaptation and are different for boys and girls, particularly when it comes to romantic and peer affiliations. Early physical maturation can lead to attachments to older boyfriends or girlfriends and exposure to social pressures and high-risk situations that younger adolescents may not be able to manage. Having older boyfriends and girlfriends is associated with early sexual activity, engagement in risky sexual practices, and enhanced risk for sexually transmitted diseases and unwanted pregnancy (Vanoss et al. 2000; Flick 1986).

For adolescent girls specifically, having an older or adult boyfriend raises the risk for underage use of alcohol and other drugs and the adoption of delinquent behaviors (Castillo Mezzich 1999). For boys, same-gender peers tend to provide more of a vector for initiation into alcohol and other drug use as well as delinquency (Kandel 1978; Dishion et al. 1994; Elliot and Menard 1996; Sampson and Laub 1993; Fergusson and Horwood 1996, 1999; Hawkins et al. 1992). Thus, boys and girls may follow different developmental trajectories, may have a different set of maturational vulnerabilities, and may have a different profile of adverse outcomes. Consequently, developmental underage drinking research needs to explore the roles of gender differences in risk and resilience.

## Multicausality, Interdisciplinary Research, and Quantitative Modeling

Multiple causal influences, from molecules to the media, interact in complicated ways over time to influence underage drinking behavior and outcomes. Building empirical models that capture this complexity can be challenging. Constructing developmental models requires repeated measurement of social, cultural, environmental, and biological factors influencing each other across time. Interdisciplinary expertise is typically required to collect and integrate these diverse types of data. Statistical models also must be developed to validly test a developmental theory—to see if it accurately reflects what happens in the real world. Although quantitative methods such as growth curve analysis—an approach to studying change over time—can address some research questions, methods have yet to be developed to deal with more complex theoretical models relevant to underage drinking and adolescent alcohol misuse (Curran and Willoughby 2003).
